# Experiences from a Community Based Substance Use Treatment Centre in an Urban Resettlement Colony in India

**DOI:** 10.1155/2014/982028

**Published:** 2014-11-09

**Authors:** Yatan Pal Singh Balhara, Rajeev Ranjan, Anju Dhawan, Deepak Yadav

**Affiliations:** ^1^Department of Psychiatry, National Drug Dependence Treatment Centre, All India Institute of Medical Sciences, New Delhi 110029, India; ^2^MSSO, National Drug Dependence Treatment Centre, All India Institute of Medical Sciences, New Delhi 110029, India

## Abstract

*Background*. There are limited community based treatment services for drug dependence in India. Rural areas and urban resettlement colonies are in particular deficient in such services. *Aims*. The current study aimed at preliminary assessment of substance use disorder management services at a community based substance use treatment clinic in an urban resettlement colony. *Methods*. The study was carried out at community based substance use treatment centre in a resettlement colony in India. The records of the centre were chart reviewed. *Results*. A total of 754 patients were registered at the clinic during the study period. Heroin was the primary drug of abuse for 63% of the patients. The mean duration of follow-up for the patients with opioid and alcohol dependence was 13.47 (SD ± 10.37; range 0–39) months. A total of 220 patients of opioid dependence were prescribed substation or abstinence directed therapy. Buprenorphine (87), slow release oral morphine (SROM) (16), and dextropropoxyphene (98) were used for opioid substitution. *Conclusion*. It is possible to deliver substance use disorder treatment services in community setting. There is a need to develop area specific community based treatment services for substance abuse in socially disadvantaged populations such as urban resettlement colonies.

## 1. Introduction

Drug use and dependence are a major public health problem globally. While tobacco and alcohol are most commonly used drugs in India, cannabis and opioids top the list of most commonly used illicit substances. As per the findings of a national survey in India, there are 62.5 million users of alcohol, 8.7 million users of cannabis, and 2 million users of opioids [1]. There is wide treatment gap for the substance users in the country [2]. Additionally most of the treatment services are centrally located.

Urbanization is a phenomenon that has become increasingly important for developed as well as developing countries over the last century [3, 4]. Consequently urban environment becomes important as a potential determinant of both health and health behavior [5]. Additionally it presents challenges with regard to meeting the health care needs of individuals inhabiting these areas.

Geographical access is an important determinant of treatment utilization in the general population [6]. Substance use treatment services have been criticized for not being easily available and accessible to those in need for such services. Longer travel distances are associated with shorter length of stay and lower probability of completion and aftercare utilization of substance use treatment services [7, 8]. Urban resettlement colony represents economically disadvantaged locality in the urban areas. People who live in economically segregated communities are likely to have disproportionately high exposure, susceptibility, and response to toxic substances and hazardous conditions [5].

Patient-centred treatment services, provided in a supportive environment and aimed at meeting the needs of the patient, result in patients remaining in treatment for longer periods of time. The “soft entry approach,” where the drug dependence treatment services were taken into community events and settings for Australian aboriginals, was found to be highly successful [9].

Importance and need of community based treatment services for substance use have increasingly been recognized in developed [10] as well as developing countries [11]. Such community based service delivery models have been found to be effective as well as financially viable [12, 13]. However, such treatment services continue to remain deficient in rural areas and urban resettlement colonies. There is a dearth of literature on substance use treatment services in urban resettlement colonies [14]. The community based camp based approach for alcohol and drug use has been documented previously in the country [15]. But these camps were based on philosophy of total abstinence. These camps did not offer maintenance therapy.

The current study presents experiences from an integrated Community Outreach Centre based substance use treatment centre in an urban resettlement colony in India. This community based substance use treatment centre was established in an urban resettlement colony. The profile of the patients attending the clinic, treatment offered, and outcomes have been discussed.

## 2. Methods

The current study was carried out at community based substance usetreatment center in a resettlement colony in India.

### 2.1. Treatment Facility

The community based services at a resettlement colony in the northern part of India were established with the aim of providing services to the local population and of developing a low cost model of treatment. This resettlement colony was established in 1975. There are five slums containing approximately 1500 to 5000 huts and a population of around 200000 within a 5 km. radius. Most of the households belong to low and lower middle income group with a high prevalence for alcohol and substance use. The substances of abuse are freely available in the locality.

During the initial phase the centre focused on increasing the treatment seeking among the substance using population and development of services in the clinic. In the subsequent years the emphasis was extended to areas like collaboration with other organizations in the area [16].

The center provides pharmacological interventions in the form of agonist maintenance (buprenorphine, morphine, dextropropoxyphene, and slow release oral morphine (SROM)). Additionally it provides services for alcohol dependence as well. The psychosocial interventions offered at the center include brief intervention, motivation enhancement, relapse prevention, sessions to enhance treatment adherence, family sessions, home visits, occupational rehabilitation, yoga workshops, recreational activities, and structuring of time. The substance use treatment centre was housed in the building of a Community Health Centre that offers general medical and surgical services to the patients in the locality.

The substance use disorder treatment clinic was set up in the premises offering Community Outreach Services. These services were being offered as an outreach project of a tertiary care institution. The health care services were delivered by the departments of community medicine, ophthalmology, and dentistry. There were no formal substance use disorders treatment services in the locality at the time of setting up of this outreach clinic.

### 2.2. Sample

All the patients that received treatment services from the treatment centre during the initial two years of service delivery were included in the study. The subjects for which treatment records were not available were excluded from the analysis. A total of 754 patients were registered at the clinic during the study period (2005-2006). Heroin was the primary drug of use for 63% of the patients. Alcohol was the second most common primary drug of use with 24% users. Other primary drugs of use were cannabis (3.9%), tobacco (3.7%), natural opium (3.5%), inhalants (0.3%), and over-the-counter medications (0.1%).

### 2.3. Measures

The current study presents the findings of the chart review of the records over a two-year period. The study presents the experiences from the initial phase of the clinic. The findings of the follow-up of the patients over a 39-month period have also been reported. The status of patients who had not made any visit in the past three months was explored using a home visit approach. Reasons for dropout of the patients from the treatment were explored.

### 2.4. Statistical Analysis

Data were analysed using licensed version of SPSS version 21 (IBM Inc., New York). Descriptive statistics were used for the sociodemographic data and drug use profile of the study subjects. Between group differences for the dropouts and retained patients student *t-*test and chi-square test for the continuous and categorical variables were carried out. The level of statistical significance was kept at *P* < .05.

## 3. Results

### 3.1. Demographic Profile

A total of 754 patients were registered at the clinic during the study period (2005-2006). The majority of the patients were male (98.01%). Sociodemographic profile of the patients has been provided in Table 1. Most of them were in the age range 31–40 years (38.5%) and 21–30 years (33.5%). The majority of the patients were illiterate (35.5%). Around sixty-three percent of the patients were married and the majority (92.69%) were living with the family. A high percentage of the patients (77.8%) were employed at the time of registration with the clinic. Around nineteen percent of them were involved in illegal activities and sixteen percent reported of having been arrested at least once.

### 3.2. Drug Use Profile

Heroin was the primary drug of use for 63% of the patients. The proportion of subjects using various psychoactive substances has been presented in Table 2.

The mean age of onset of use of primary drug of use was 22.8 (SD ± 7.38; range 8–50) years. The mean duration of regular use of the primary drug of use was 10.7 (SD ± 8.04; range 1–55) years. The majority of the subjects (39.54%) had used the drugs for more than 10 years. Only 6.97% of the patients had used them for less than one year. 17.47% of the patients reported history of injecting drug use ever. History of a prior abstinence attempt was given by 40.48% of the patients and the majority (60.4%) had not received any treatment in the past.

### 3.3. Treatment Retention and Dropouts

The proportion of female patients was higher in the dropout after first visit group as compared to the retained group (4.3% v/s 0.8%, *P* < .001). No significant differences were observed between those dropped out after the first visit and those enrolled for long term treatment on other sociodemographic variables. The proportion of opioid dependent subjects was higher in the retained group as compared to the dropout group (72.8% v/s 43.3%, *P* < .001). However, the proportion of alcohol dependent subjects was higher in the dropped out group as compared to the retained group (35.4% v/s 18.2%, *P* < .001).

The mean duration of follow-up for the patients with opioid and alcohol dependence was 13.47 (SD ± 10.37; range 0–39) months. 254 (33.7%) of the patients dropped out after the first visit. 46.15% of the patients with opioid dependence followed up for more than six months, 13.74% for a period less than six months but more than one month, and 40.11% followed up for less than a month. Similarly, 67.03% of alcohol dependent patients followed up for a period more than six months. 9.89% followed up for a period less than six months but more than one month and 23.98% followed up for less than a month. 57.46% of opioid dependent subjects reported abstinence during last month of follow-up as compared to 63.22% of alcohol dependent patients.

A total of 220 patients of opioid dependence were prescribed substation or abstinence directed therapy. Buprenorphine (39.54%), slow release oral morphine (SROM) (7.27%), and dextropropoxyphene (44.54%) were used for opioid substitution. Nineteen patients were put on naltrexone. Disulfiram was prescribed to 11 patients for alcohol dependence. Among the patients with opioid dependence 3-month compliance rate was 86.4% for buprenorphine, 87% for SROM, and 66.1% for naltrexone. The compliance rates on buprenorphine and SROM were significantly higher than that for naltrexone (*P* < .05).

An attempt was made to explore the status of patients who had not made any visit in the past three months. A total of 42 such patients were identified and visits were made to their home. Reasons for dropout have been presented in Table 3.

## 4. Discussion

The study reports experience from a community based substance use treatment centre in an urban resettlement colony. The profile of the patients attending the clinic, treatment offered, and outcomes have been explored. Almost all the treatment seekers in the current study were young males. This partly reflects the higher prevalence of drug use problems among males. Most of the drug users in India are male but in many drug treatment centers female drug users may constitute up to 10% depending on the city and geographic region [17]. However the stigma associated with female drug use is likely to have contributed to the lower percentage of female patients at the treatment centre. Drug and alcohol use prevalence has been found to be highest among young adults. In fact, in a recent survey on drug users, 15% respondents were female [18].

Community based substance use services have been recommended to ensure better service utilization [12, 13]. It has been reported from western settings that community settings often possess considerable resources in the form of local community based organizations (CBOs), which have extensive ties both within the neighborhood and throughout the broader community. Also, it has been recommended that the intervention programs may be more narrowly tailored and targeted in terms of problems addressed, general strategies, and particular tactics using community based intervention models [19].

A high proportion of treatment seekers were married, were living with family, and were employed. This provides a unique opportunity to involve the family members in the treatment process. Also it brings in the need to include appropriate support services for the family members. Another important observation with implications on service delivery was that of suitability of dispensing hours for the medications. Since a large number of treatment seekers were employed the daily dispensing of opioid substitution therapy was particularly challenging.

Heroin was the most common primary drug of use. This is an interesting finding as the previous studies from India have reported alcohol as the most common primary drug in community based as well as treatment centre settings [20–23]. A prior national survey in treatment settings in the country reported prevalence of opioid use to be 26.0% among treatment seeking population [1].

An Indian study reported that in urban slum population both the ages at first use and regular use of alcohol and substance were earlier than the rural population. Alcohol was the most commonly used substance reported in this study [14]. The mean age of first use of the primary drug in the current study (22.8 years) was higher than the mean age of first use reported in this study (19.75 years). Also the duration of use (10.7 years) found in the current study was lower than that reported in this study (24.12 years).

The majority of the patients coming to the treatment centre had not received any prior treatment. This could be explained by lack of community based services in the area. Moreover, the poor economic status of the residents would have precluded out of pocket spending on treatment. Low rate of treatment seeking was also reported in the urban slum based Indian study [14].

A higher proportion of opioid dependent patients were retained in the treatment. This was due to availability of various substitution agents (buprenorphine, dextropropoxyphene, and SROM) for opioid dependence at the centre. A high proportion of opioid dependent patients were put of OST. On the other hand, the only long term treatment available for alcohol was a deterrent therapy in the form of disulfiram. A previous study from UK reported that alcohol was the only substance significantly associated with treatment retention, with alcohol users less likely to drop out of treatment than nonalcohol users [24].

The three-month compliance rates of 86.4% for buprenorphine, 87% for SROM, and 66.1% for naltrexone were very high. Previous studies have reported the three-month compliance rate on opioid agonists to be around 60% [25]. The three-month compliance rates for naltrexone have been found to be around 33% [25]. However, it should be kept in mind that no urinalysis could be performed to confirm presence of treatment medication as well as drugs of use. It was so because no laboratory services were present at the centre in the initial phase.

A high follow-up rate for both opioid and alcohol dependent patients was achieved. The mean duration of follow-up observed in the current study if higher than that reported in community based treatment services. Close proximity of the treatment facility to the place of residence of the patients is a likely reason for this high retention rate observed in the current study. Substance use treatment services like National Treatment Agency for Substance Misuse (NTA) in UK aim at retaining 75% of individuals for 12 weeks or more during each annual reporting cycle [26]. Individuals not retained in treatment for at least three months were unlikely to experience long term benefits [27]. Patients who remain in treatment have better treatment outcomes due to adherence compliance and persistence [28, 29]. Research strongly suggests that length of time spent in treatment (or treatment retention) is the single most consistent predictor of successful AOD treatment outcomes [30–32]. Retaining drug users in treatment has been recommended as an important intermediate goal of the treatment system. In fact, retention in drug treatment is considered by the NTA to be the best available measure of treatment effectiveness [33]. While some studies have suggested that younger drug users are more likely to drop out of treatment [31, 34], others have failed to find such association [35]. The duration of use of psychoactive substances was relatively lower in the current study.

The most common reason of dropout from the treatment in the current study was being incarcerated. A high proportion of patients had history of involvement in crime at the time of enrollment in the programme. However most of the opioid dependent patients dropping out of treatment reported abstinence when approached in their homes. The Indian survey in urban slum found that many of the substance users are not motivated toward help seeking and hence required treatment delivery at their doorstep [14]. Studies from the west have found that individuals referred from the criminal justice system are significantly more likely to drop out and less likely to complete treatment drug free than those referred from noncriminal justice sources [24].

The current study presents the experiences from a community based drug dependence treatment centre in an urban resettlement colony. There are no published reports on such treatment services from India. The community based camp based approach for alcohol and drug use has been documented previously in the country [15]. But these camps were based on philosophy of total abstinence. These camps did not offer maintenance therapy. Multiple treatment options were offered to the opioid dependent patients. The services were, however, limited for alcohol dependent patients. Home visits were made to contact the treatment dropouts. We had encouraging findings on chart review of the service utilization at the centre.

## 5. Implications for Behavioral Health

These findings suggest the feasibility of setting up community based drug dependence treatment services in the country. Longer travel distances are associated with shorter length of stay and lower probability of completion and aftercare utilization of substance use treatment services [7, 8]. Distance may be an especially relevant factor among patients who live in areas with poor public transportation facilities. Opioid dependent patients who live at a greater distance from a treatment centre are likely to be at higher risk for dropping out of treatment than patients who live closer to a program [36]. Taking treatment services at the doorsteps of drug users helps overcome the barriers of accessibility and affordability of treatment services.

The findings of the current study have important clinical and policy implications. The findings of the current study provide insight into the profile of the individuals seeking treatment from a community based substance use treatment centre in an urban resettlement colony. There is limited literature on this issue. Urbanisation is a global phenomenon. It has impacted both the developed and the developing countries [3, 4, 11]. It has been seen that substance use problems tend to initiate in the metropolitan subpopulations before they spread to the other areas in a country [5]. Within the urban areas the people living in economically disadvantaged communities like urban resettlement colonies have disproportionately higher exposure, susceptibility, and response to toxic substances and hazardous conditions [37]. Urban characteristics, social networks/psychosocial stressors, and area characteristics/living conditions are three important mediators of the substance use problem in urban areas. Urban resettlement colonies are in a position of disadvantage on account of all of these three factors. Specific structural conditions of concentrated poverty have been found to be more salient than race in explaining the violence and substance use nexus in American urban areas [38, 39]. These low income urban communities are characterised by poverty, joblessness, welfare dependency, and interlocking social problems [40].

Hence it is important to establish appropriate substance use treatment services in such localities. Availability of regular, good-quality medical care may contribute to lower prevalence of drug use in urban areas [41].

There is a need to develop area specific community based treatment services for substance use in socially disadvantaged populations such as urban resettlement colonies. Use of integrated care models where substance use treatment services are provided along with the general medical care through the community health centers is likely to be of even greater benefit [10].

### 5.1. Strengths and Limitations

The current study is the first report on findings from a community based substance use disorder treatment centre in an urban resettlement colony in the country. There is limited literature on this issue from India. All the subjects seeking treatment from the centre in the first two years were included in the study. An attempt was also made to contact the treatment dropouts. However, the study has certain limitations as well. We did not carry out cost-effectiveness analysis of the services offered at the centre. Additionally, the outcome was not assessed using a fully structured questionnaire.

### 5.2. Future Directions

However these are preliminary findings. There is a need to review the records at periodic intervals to assess the trends over time. There is a need to augment treatment services for alcohol dependent individuals. The future studies must incorporate structured questionnaires to assess the outcome of the study subjects. Also, it shall be helpful to use urinalysis based objective measures of treatment adherence. Moreover, the model needs to be replicated in other areas of country to assess its feasibility and effectiveness. Also structured studies need to be carried out to assess cost-effectiveness of such interventions in community setting.

## Figures and Tables

**Table 1:** Sociodemographic profile of the subjects.

Variable	Percentage
Gender	
Males	98.01
Females	1.99
Age	
16–20 years	7.39
21–30 years	33.55
31–40 years	38.2
41–50 years	15.65
51 years and above	5.31
Educational qualification	
Illiterate	35.54
Literate	5.84
Primary education	20.56
Middle school	19.89
Up to secondary/senior secondary	15.25
Graduate	2.79
Postgraduate	0.13
Marital status	
Never married	25.2
Married	65.92
Widow/widower	5.31
Separated	3.5
Living with family	
Yes	92.69
No	7.31
Employment status	
Employed	77.8
Never employed	12.5
Previously employed	9.7

**Table 2:** Psychoactive substance used by the study subjects.

Psychoactive substance	Number	Percentage
Heroin	472	63.0
Opium	26	3.5
Other opiates	11	1.5
Alcohol	180	24.0
Cannabis	29	3.9
Tobacco	28	3.7
Inhalants	2	0.3
Over-the-counter drugs	1	0.1

**Table 3:** Reasons for dropout from treatment among study subjects.

Reason for dropout from treatment	Percentage
Shift in residence	23.8
Being in jail	9.5
Death	4.8
Moving away from home without any information	7.1
Could not be ascertained	54.8
